# MicroRNA-30c-2-3p regulates ER stress and induces apoptosis in ovarian cancer cells underlying ER stress

**DOI:** 10.17179/excli2020-2970

**Published:** 2021-05-25

**Authors:** Shekufe Rezghi Barez, Ahmad Movahedian Attar, Mahmoud Aghaei

**Affiliations:** 1 Department of Clinical Biochemistry, School of Pharmacy & Pharmaceutical Sciences, Isfahan University of Medical Sciences, Isfahan, Iran

**Keywords:** miR-30c-2-3p, XBP1, ovarian cancer, ER stress, apoptosis

## Abstract

Ovarian cancer is a common gynecologic cancer with a high rate of recurrence, drug resistance, and mortality, thereby necessitating novel molecular target therapies. Ovarian cancer as a solid tumor has constantly been challenged by endoplasmic reticulum stress (ERS). Currently, XBP1 as a therapeutic target in solid tumors plays a key role in adaptation to ERS. Single-stranded RNAs usually modulate posttranscriptional of the gene activity. miR-30c-2-3p has been demonstrated to inhibit the expression of XBP1. Here, we evaluated the effect of miR-30c-2-3p on controlling XBP1-CHOP-BIM and its apoptotic effects on ovarian cancer cell lines during ERS. The ER stress was assessed using Thioflavin T staining in OVCAR3 and SKOV3 cells. The expression of ER stress genes was measured by QRT-PCR. The protein levels of XBP1(s), BIP/GRP78, CHOP, and BIM were evaluated using Western blotting. Cell viability and apoptosis in STF-083010 and Tunicamycin (Tm) co-treated cells were evaluated using BrdU, MTT, Annexin V-FITC/PI staining, and caspase-12 and -3 activities assays. We found that miR-30c-2-3p significantly decreased the folding capacity of ER, leading to ERS intensification (P<0.05). Additionally, the Western blot analysis showed the modest up-regulation of CHOP and BIM with pro-apoptotic activity and down-regulation of the BIP protein. Furthermore, mimic miR-30c-2-3p transfection not only decreased cell proliferation but also induced cell death in ovarian cancer cells in response to the Tm-treatment. Our results indicated that the apoptotic pathway was induced possibly through activation of caspases -12 and -3 and elevation of the Bax/Bcl-2 ratio. Overall, the present paper adds new evidence to the possible treatment of miR-30c-2-3p via impeding the XBP1 transcription in ovarian cancer cells provoking apoptotic pathways by XBP1/CHOP/BIM mediators.

## Introduction

XBP1 is a key adaptive factor of unfolded protein response (UPR) signaling that is stimulated in response to endoplasmic reticulum (ER) stress (Blazanin et al., 2017[6]). The ER stress activates IRE1α (inositol-requiring kinase1) that is an inducer of UPR. Then, the endoribonuclease activity of IRE1α cuts a 26 nucleotide intron from XBP1 mRNA to generate a highly active transcription factor spliced XBP1 (XBP1s) (Bagheri-Yarmand et al., 2019[3]). The sXBP1 protein is a member of the basic-leucine zipper family interacting with the sequence-specific DNA and encodes various genes involved in restoring the ER homeostasis and survival (Das et al., 2008[11]; Jindrich and Degnan, 2016[18]). Importantly, XBP1s induces the chaperone gene expression enhancing the ER folding capacity of mis/unfold proteins (Lee et al., 2003[20]). Moreover, XBP1s are involved in glucose and lipid metabolism, immune cell differentiation (Cubillos-Ruiz and Glimcher, 2016[10]; Shi et al., 2019[30]), cancer cell metastasis, angiogenesis, and autophagy (Jindrich and Degnan, 2016[18]; Margariti et al., 2013[23]; Xia et al., 2016[42]). Many studies have demonstrated a positive correlation between the IRE1α-XBP1 activity and the pathologic condition in different diseases, particularly ovary tissue (Koelwyn et al., 2015[19]).

Several studies have reported the activation and function of IRE1α-XBP1 in ovary tissue and related diseases. A high expression level of XBP1 has recently been reported in polycystic ovarian syndrome and ovarian hyper-stimulation syndrome (Okamura et al., 2017[25]; Takahashi et al., 2016[36]). XBP1s creates dendritic cell dysfunction and defects anti-cancer immunity in ovarian cancer (Song et al., 2018[34]). Therefore, some studies have abrogated the XBP1 expression by single-stranded RNAs. For example, Wang et al. targeted XBP1 by RNAi. They showed that knockdown of XBP1 could promote apoptosis, inhibit cell cycle, and reduce estradiol synthesis in mouse granulosa ovary cells (Wang et al., 2017[41]). Zhang et al. down-regulated XBP1 by shRNA and showed that it reduced ovarian cancer cell viability and enhanced sensitivity to oxidative stress by increasing intracellular ROS levels (Zhang et al., 2019[43]). These studies suggest that XBP1 may be a potential target for ovarian cancer therapy. 

MicroRNAs (miRNAs), short noncoding RNAs (18-25 Nt), regulate the gene expression that exhibits potential clinical relevance in the regulation of cancer-related genes and is used for targeting therapy (Deb et al., 2018[12]). Recently, it has been demonstrated that miR-30c-2-3p controls the XBP1 expression. NF-κB, as the downstream of PERK, binds to the upstream motif of miR-30c-2-3p in the activated UPR, causing the induction of the miR-30c-2-3p expression in HeLa cells (Byrd et al., 2012[7]). In addition, miR-30c-2-3p can inhibit the Bcl-9 expression and suppress growth factors in response to the LPA stimulation in ovarian cancer cells (Jia et al., 2011[16]). Nevertheless, miR-30c-2-3p studies are inconsistent. In this study, we investigated the role of mimic miR-30c-2-3p in XBP1(s) targeting and ERS signaling. We also evaluated the apoptosis rate in OVCAR3 and SKOV3 malignancy cell lines during the ER stress in the presence of mimic miR-30c-2-3p.

## Materials and Methods

### Cell culture and reagent

The assessment was entirely conducted on two ovarian tumor cell lines. The OVCAR3 (C430) and SKOV3 (C209) cells were received from the National Cell Bank of Iran (NCBI, Pasteur Institute of Iran), Tehran, Iran. The cells were cultured in an RPMI-1640 medium and under standard conditions, as recommended by NCBI. The cell culture media and supplements, such as RPMI1640 medium, fetal bovine serum (FBS), penicillin and streptomycin, and trypsin/EDTA solution, were obtained from Gibco (Life Technologies GmbH, Karlsruhe, Germany). Tunicamycin (Tm; PubChem CID: 56927832) was obtained from Sigma‐Aldrich Merck (St. Louis, MI) Company.

### microRNA mimic transfection

The mimic miR-30c-2-3p, which was sourced by Sigma (MISSION® microRNA Mimic HMI0461), was used to transfect cell lines. Transfection was performed with Lipofectamine 2000 (Santa Cruz) based on the manufacturer's instructions as described previously (Li et al., 2013[21]). Briefly, OVCAR3 or SKOV3 cells (2x10^5^) were seeded into a 6-well plate and starved in a free antibiotic medium overnight. Then, the cells were transfected with mature miR-30c-2-3p (50 ng/ml) (CUGGGAGAAGGCUGUUUACUCU) and scrambled control RNAs as a negative control in 6 µl Lipofectamine 2000 and diluted by a transfection medium. After 6 hours, the medium was exchanged with an RPMI-1640 (FBS 15 %) medium and starved under standard conditions.

### mRNA gene expression

To confirm transfected miRNA and evaluate ER stress markers after the Tm treatment (Table 1(Tab. 1)), QRT‐PCR was accomplished. Briefly, 2x10^5^ cells were seeded in triplicate in 6-well tissue culture plates. After one full night, the cells were transfected with mimic miR-30c-2-3p. Twenty-four hours after the transfection, the cells were treated with Tm (3 µg/ml) for 18 hours. Total RNA was isolated and converted to cDNA as a template, using the first-strand cDNA synthesis kit (Takara Shuzo, Otsu, Japan). Real-time PCR was performed using High ROX MIX Green (AMPLIQON). Table 1(Tab. 1) lists the sequences of the primers. GAPDH was measured as the internal control, and it normalized the amount of the total RNA present in each reaction.

### Cytotoxicity assay

MTT assays were performed to assess the percentage of viable cells in the presence of miR-30c-2-3p. In brief, 10x10^3^ cells/well were seeded in 96-well cell plates. After overnight, mimic Mir30c-2-3p was transfected and starved under standard conditions for 24 hours. Then, the transfected cells were incubated with Tm (3 µg/ml). After an 18-hour incubation period, the cells were exposed to 5 mg/ml MTT dye for 4 hours. Finally, formazan crystals were dissolved in DMSO (200 µl/well) to produce a final reaction color. The absorbance of the MTT reaction color was measured in 570 nm using a Multimode Microplate Reader. The viability of control cells was assumed at 100 %.

### Bromodeoxy uridine cell proliferation assay

BrdU assay was performed to evaluate the effect of mimic miR-30c-2-3p on the cell growth by an anti-BrdU antibody as described previously (Azizi et al., 2019[2]). The incorporation rate of BrdU into DNA during cell proliferation showed the rate of the cell growth. Briefly, the cells were seeded in 96-well plates (5x10^3^ cells/well) at night. Transfection of mimic miR-30c-2-3p was started the next day. Twenty-four hours after transfection, the cells were exposed to Tm (3 µg/ml) for 18 hours. Then, the BrdU solution was added (10 μL/well) and incubated with the Fixing/Denaturing solution. After the incubation time, a secondary HRP antibody and an HRP substrate were added to develop a final reaction color. Finally, a stop solution (1 M) was added, and the absorbance was read at 370 nm.

### Thioflavin T (Th T) staining assay

Thioflavin T (ThT; PubChem CID: 16953) was provided from Sigma‐Aldrich Merck (St. Louis, MI) Company. ThT, bound to aggregated proteins, could detect endoplasmic reticulum stress after exposing cells to inducers (Beriault and Werstuck, 2013[5]). The cells (5x10^3^) were cultured in 96-well plates, and then, the cells were transfected with mimic miR-30c-2-3p for 24 hours. Afterward, the cells were treated with Tm (3 µg/ml) for 18 hours. After washing with PBS, the cells were embedded by paraformaldehyde 4 % for 20 minutes. After fixation, 5 μl of 1 mM Thioflavin T (Th T) was added to the wells and incubated. The cells were then washed with PBS, and fluorescent images of living cells were taken with a Zeiss Axioplan 2 microscope. The fluorescence intensity was read at 485 nm excited with a blue dye filter, and the images were acquired at 535 nm emission under 10 fields of view and semi-quantified by the Image J software.

### Western blots and antibodies for protein detection

The ER stress proteins level (XBP1, sXBP1, BIP, BIM, CHOP, Bax, and Bcl-2) was assessed using Western blotting. The cells were seeded in 6-well plates, and mimic miR-30c-2-3p was transfected. The following day, the cells were incubated with Tm (3 µg/ml) for 18 hours. After the incubation, the cells were lysed, and the protein concentration was measured using the Bradford Protein Assay. In addition, a total of 20-30 µg of each protein sample was loaded on the wells of SDS-PAGE to be separated and transferred into polyvinylidene difluoride (PVDF) membrane (Amersham Pharmacia Biotech, Buckinghamshire, United Kingdom). After blocking the process and incubating it with primary monoclonal antibodies, including XBP1 1:200 (F-4: sc-8015), sXBP1 1:500 (12782S), CHOP 1:500 (9956T), BIM 1:200 (sc-374358), BIP 1:500 (9956T), Bax 1:200 (sc-7480), Bcl-2 1:200 (sc-492), and B-actin 1:200 (sc-47778) as the housekeeping antibody, the membrane was washed three times in TBST and incubated by conjugated secondary antibodies 1/5000 for 100 minutes (Santa Cruz Biotechnology). The band of proteins was revealed by ECL (Amersham Pharmacia Biotech, Buckinghamshire, United Kingdom) and visualized using a Doc Print CX5 Gel Documentation system (Vilber Lourmat) to produce digital images. Finally, these images were semi-quantified by the Image J software. The level of proteins was normalized by B-actin (C4: sc-47778).

### Detection of cell death using Annexin V/PI staining

The programmed cell death was measured by the Annexin V-FITC/PI kit (Biovision). Briefly, the cells were grown in plates (6 wells) to reach 70 % confluence, and then miR-30c-2-3p was transfected into OVCAR3 and SKOV3 cells. After 24 hours, the cells were exposed to Tm (3 µg/ml) for 18 hours. Finally, the cells were collected, and the procedure was performed as previously described (Azizi et al., 2019[2]). The results were obtained by a FACS Caliber flow cytometer (BD Bioscience) and the FlowJo software.

### Caspases -12 and -3 fluorometric activities assay

Caspase-12 and -3 activities were evaluated by the fluorometric method according to the manufacturer's instructions (Biovision Systems, USA) as described previously (Arasi et al., 2019[1]). Shortly, the cells (5x10^5^) were cultured overnight in a culture plate, and mimic miR-30c-2-3p was transfected into cells for 24 hours. Then, Tm (3 µg/ml) was added and incubated for 18 hours. Afterward, the cells lysed by lysis buffer and supernatants were collected. The fluorescence substrates ATAD-AFC (for caspase-12) and DEVD-AFC (for caspase-3) were added to the samples and incubated for 2 hours. Finally, the samples were transferred in a 96-well flat bottom microplate, and fluorescence was read at 400/505 nm ex/em using a Microplate Reader (Synergy H1 Hybrid MultiMode BioTek). The results were shown as fold changes to be compared to the scramble.

### Statistical analysis

All the data were processed and analyzed using Graph Pad Prism. The results presented as the mean ± SD as the average of independent triplicate experiments (with 3 time repeats). A statistical non-parametric one-way analysis of variance (ANOVA) test followed by Tukey Post-hoc test to compare data and P value < 0.05 was considered statistically significant.

## Results

### MicroRNA-30c-2-3p impeded XBP1 mRNA expression 

**Figure 1:**
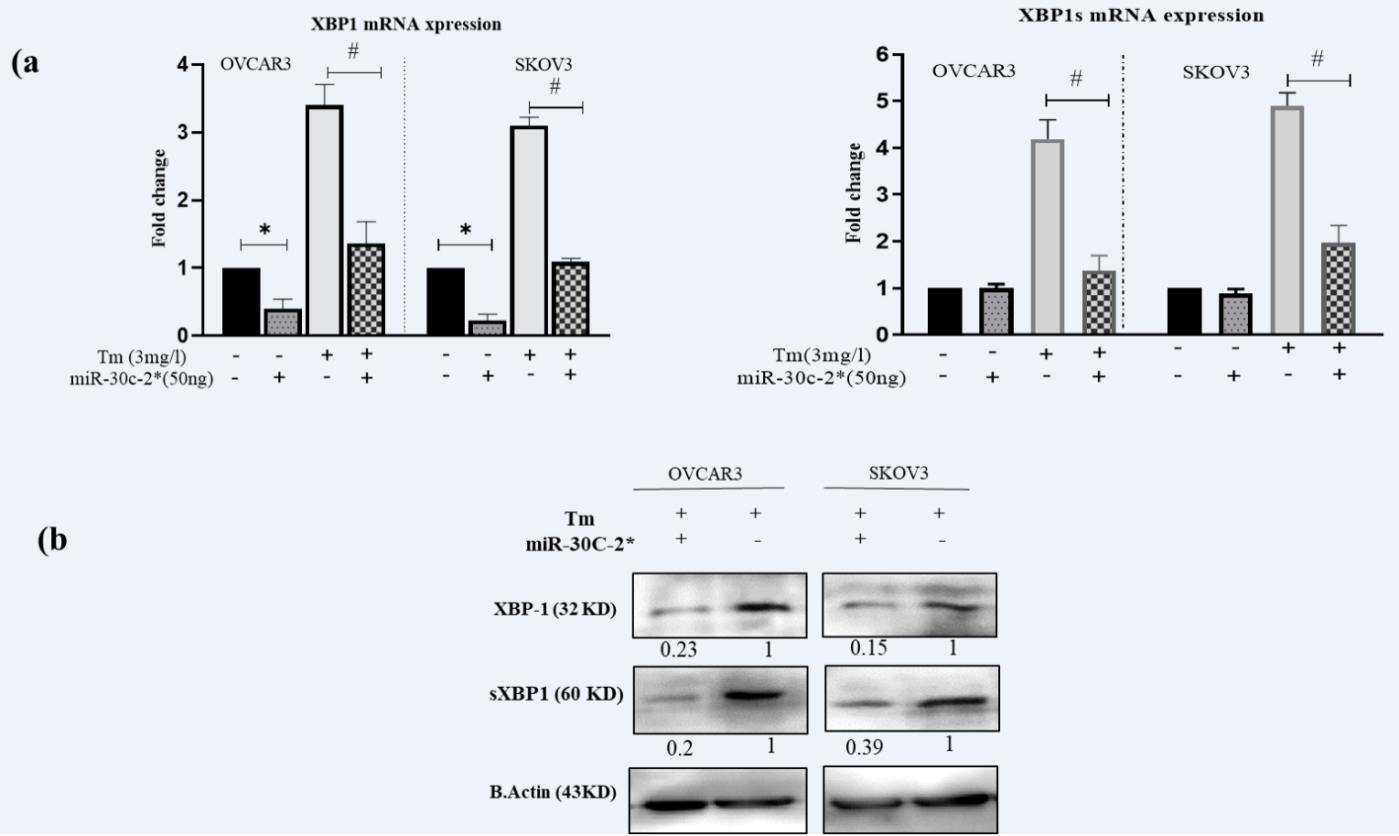
Figure 1: Transfection of miR-30c-2-3p reduced the expression levels of XBP1 and sXBP1 (mRNA and protein) in the cells during ER stress: a) The expression levels of XBP1/s mRNA in transfected and non-transfected cells in presence Tunicamycin, b) Digital imaging of Western blot detection of XBP1/s protein levels in two ovarian cancer cell lines. Beta-actin was loaded for data normalization and Semi-quantitative analysis was made by Image J software. Representative blots were duplicated in three independent experiments. Results were presented as mean ±SD and p value<0.05 (*) compared with untreated cells (control) and p<0.05 (#) compared with Tm-treated cells considered as significant.

To examine the regulatory capacity of miR-30c-2-3p on the XBP1 expression in ovarian malignancy cells, we transfected OVACR3 and SKOV3 by miR-30c-2-3p. Moreover, the transfected and non-transfected cells were exposed to Tunicamycin inducing the ER stress by the inhibition of N-linked glycosylation, and then the XBP1/s mRNA expression was evaluated using RT-PCR (Figure 1a(Fig. 1)). The XBP1 expression was lower in miR-transfected cells compared to the scramble (P˂0.05) and Tm treated (P˂0.05) cells. The ER stress activates IRE1α that cleavages XBP1 mRNA and generates sXBP1. The expression of XBP1s was reduced by the transfection of miR-30c-2-3p, when compared to the Tm treatment (P˂0.05). We also measured the protein expression of both proteins (XBP1 and sXBP1) by Western blotting. The results showed that the XBP1 and sXBP1 expressions were considerably decreased in transfected cells, and XBP1 and sXBP1 expressions were semi-quantified 0.23 and 0.2 in OVCAR3, and 0.15 and 0.39 in SKOV3 cells, as illustrated in Figure 1b(Fig. 1).

### MicroRNA-30c-2-3p regulated the ER stress in ovarian cancer cells

**Figure 2:**
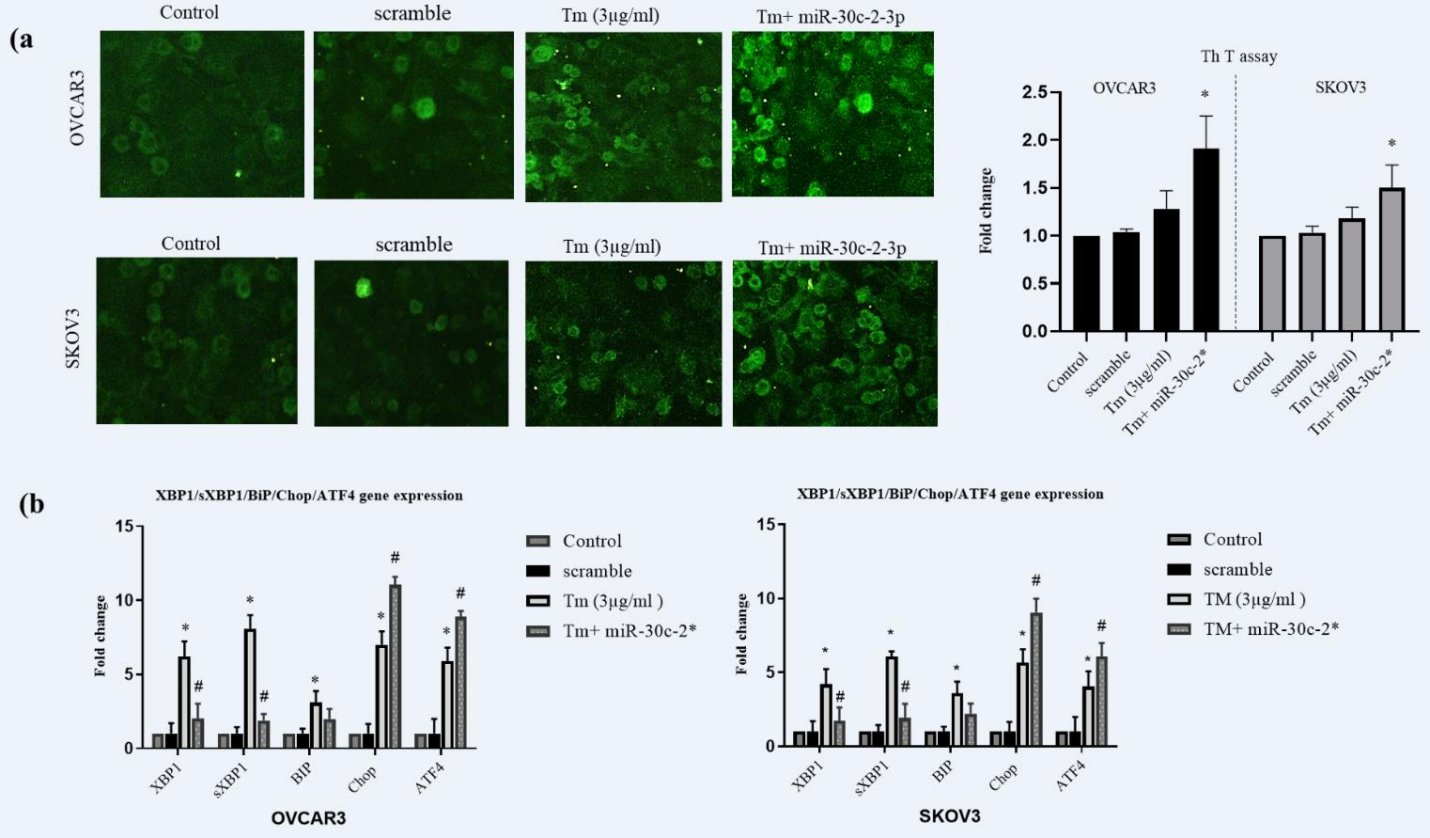
Transfection of miR-30c-2-3p increased ER stress via reduction of XBP1s level. (a) ThT staining was performed on cells and images were captured by microscope (Zeiss Axioplan 2). (b) The expression levels of BIP, CHOP, ATF4, XBP1, and sXBP1 mRNA were evaluated by real-time RT-PCR method (triplicated samplings). The results were manifested as mean ±SD and p<0.05 (*) and p<0.01 (**) compared with untreated cells (control) and p<0.05 (#) compared with Tm-treated cells considered as significant.

It is demonstrated that XBP1 encodes several ER chaperons and their regulatory factors. Therefore, we assessed the intensity of the ER stress in miR-30c-2-3p transfected cells by the Thioflavin T staining assay (ThT). ThT as a fluorescent agent binds to aggregated proteins and visualizes the endoplasmic reticulum stress. We found that transfected cells showed (Figure 2a(Fig. 2)) modestly higher stimulation of the ER stress in response to the Tm treatment (1.51±0.34 fold in OVCAR3 and 1.25±0.22 in SKOV3 cells compared to Tm-treated cells). 

To further study the capacity of the miR-30c-2-3pin regulation of the ER stress, we evaluated the expression of ER stress XBP1, sXBP1, BIP, CHOP, and ATF4 genes using RT-PCR. The results indicated that the ER stress gene expression was altered in transfected cells in response to the Tm treatment shown in Figure 2b(Fig. 2). These results revealed that inhibition of XBP1 through miR-30c-2-3p modestly increased the Tm‐mediated protein aggregation and provoked the ER stress in both ovarian cancer cell lines.

### MicroRNA-30c-2-3p induced apoptosis against ovarian cancer cells underlying the ER stress

**Figure 3:**
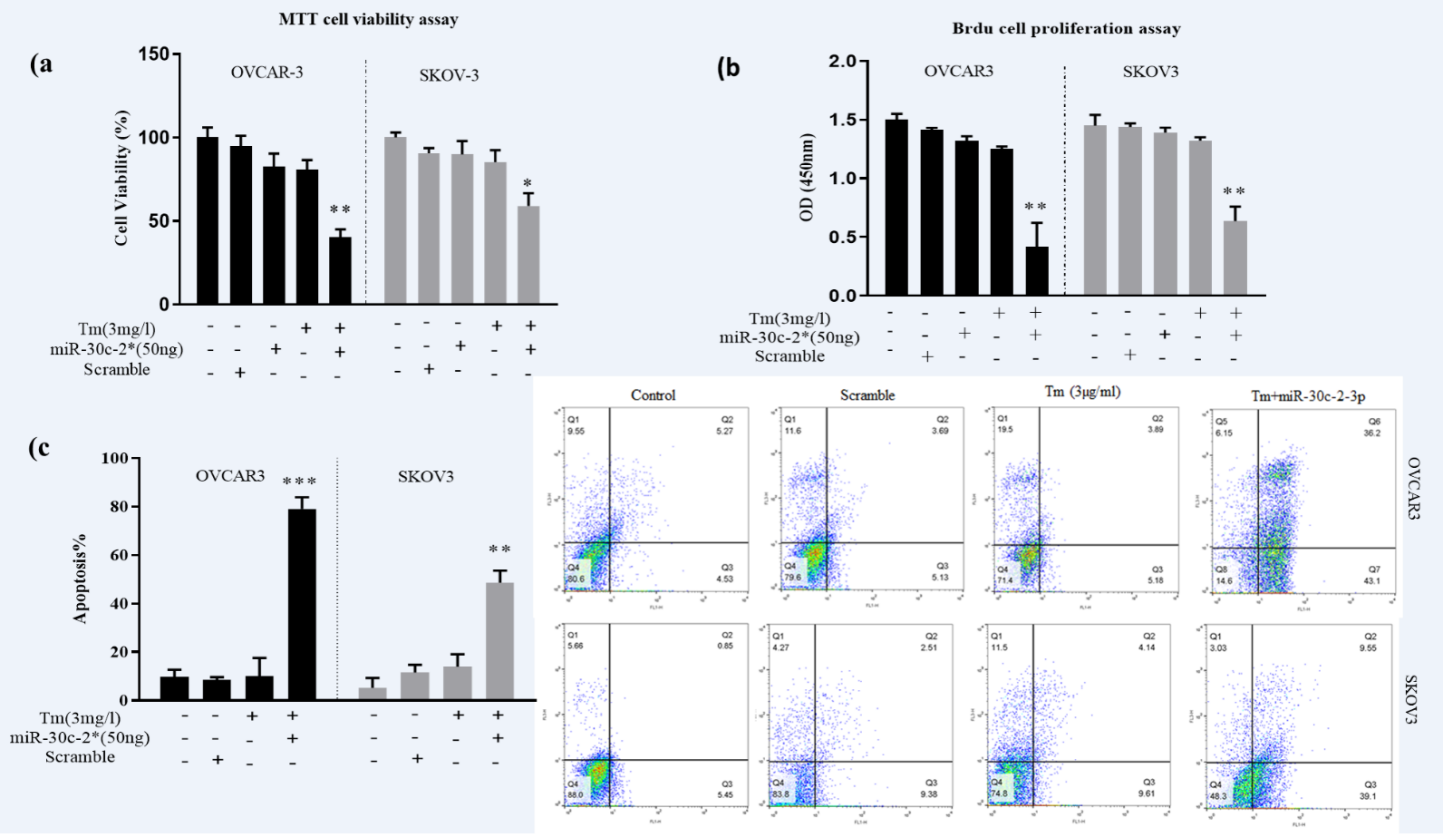
Transfection of miR-30c-2-3p promoted ER stress-induced cell death and decreased viability percentages in ovarian cancer cells. OVCAR3 and SKOV3 were transfected by miR-30c-2-3p and treated with Tm, and then, the viability, proliferation and apoptosis of cells were studied via (a) MTT, (b) BrdU assays, and (c) Annexin-FITC/PI analysis, respectively. All analyses were made in triplicate, and each case repeated three times (n=9). Results were presented as mean ± SD and p<0.05 (*), p<0.01 (**) and p<0.001 (***) considered as statistically significant.

To examine the effects of miR-30c-2-3p on the growth of the cells through the ER stress, we performed MTT and BrdU assays. Briefly, mimic miR-30c-2-3p was transfected in two ovarian cancer cell lines (SKOV3 and VOCAR3), then exposed to the Tm treatment for 18 hours. The cell viability was significantly decreased by mimic miR-30c-2-3p in the presence of the Tm treatment (40±5.6 % in OVCAR3 cells; p<0.01. 59±7.52 % in SKOV3; p˂0.05, compared to un-transfected cells) in both cell lines (Figure 3a(Fig. 3)). Moreover, the results of the BrdU (Figure 3b(Fig. 3)) assay revealed that microRNA-30c-2-3p could remarkably inhibit proliferation in both ovarian cancer cell lines (P˂0.01). To further confirm the apoptotic effects of microRNA-30c-2-3p, we evaluated cell death by the flow cytometry analysis. The percentage of apoptosis was 79.3±5.8 % in OVCAR3 and 48.65±4.5 % in SKOV3 transfected cells (Figure 3c(Fig. 3)).

### MicroRNA-30c-2-3p up-regulated proteins associated with apoptosis during the ER stress 

**Figure 4:**
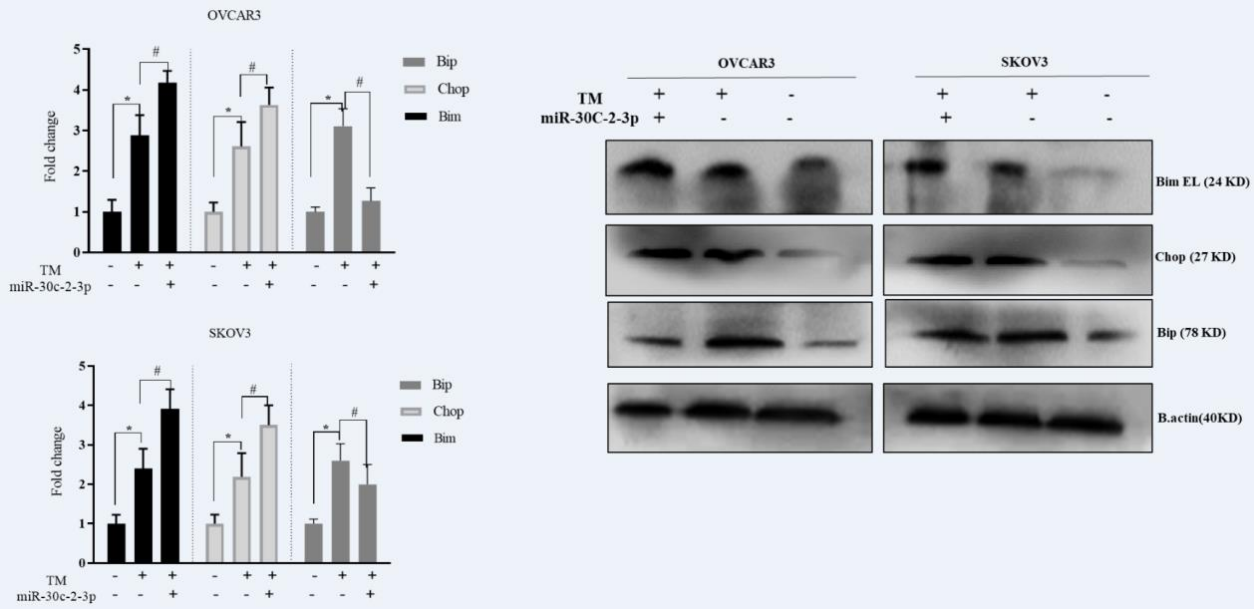
Blockage of XBP1 via miR-30c-2-3p modestly induced apoptosis-associated proteins in ovarian cancer cell lines. Western blot detection of CHOP, BIM and BIP/ GRP78 in OVCAR3 and SKOV3 after exposure with Tm for 18 h. Beta-actin used as a loading control and Semi-quantitative analysis was carried out by Image J software. Results were presented as mean ± SD and p<0.05 (*) in comparison with the control, and p<0.05 (#) in comparison with Tm-treated cells considered as significant.

In the next step, we found that the blockage of XBP1 via microRNA-30c-2-3p could influence the CHOP, BIM and BIP/GRP78 chaperon in response to the Tm treatment. The cells were transfected with microRNA-30c-2-3p and undergone the Tm treatment. The transfection of MicroRNA-30c-2-3p induced the CHOP and BIM protein expression in response to the Tm treatment (1.25 and 1.38 fold for transfected OVCAR3 cells, 1.27 and 1.22 fold for transfected SKOV3 cells, respectively). However, the level of the BIP protein was decreased (Figure 4(Fig. 4)). These results showed that mimic MicroRNA-30c-2-3p could induce pro-apoptotic proteins associated with the ER stress in ovarian cancer cells in the presence of the Tm treatment. 

### MicroRNA-30c-2-3p influenced caspase-3 and -12 activities and Bax/Bcl-2

**Figure 5:**
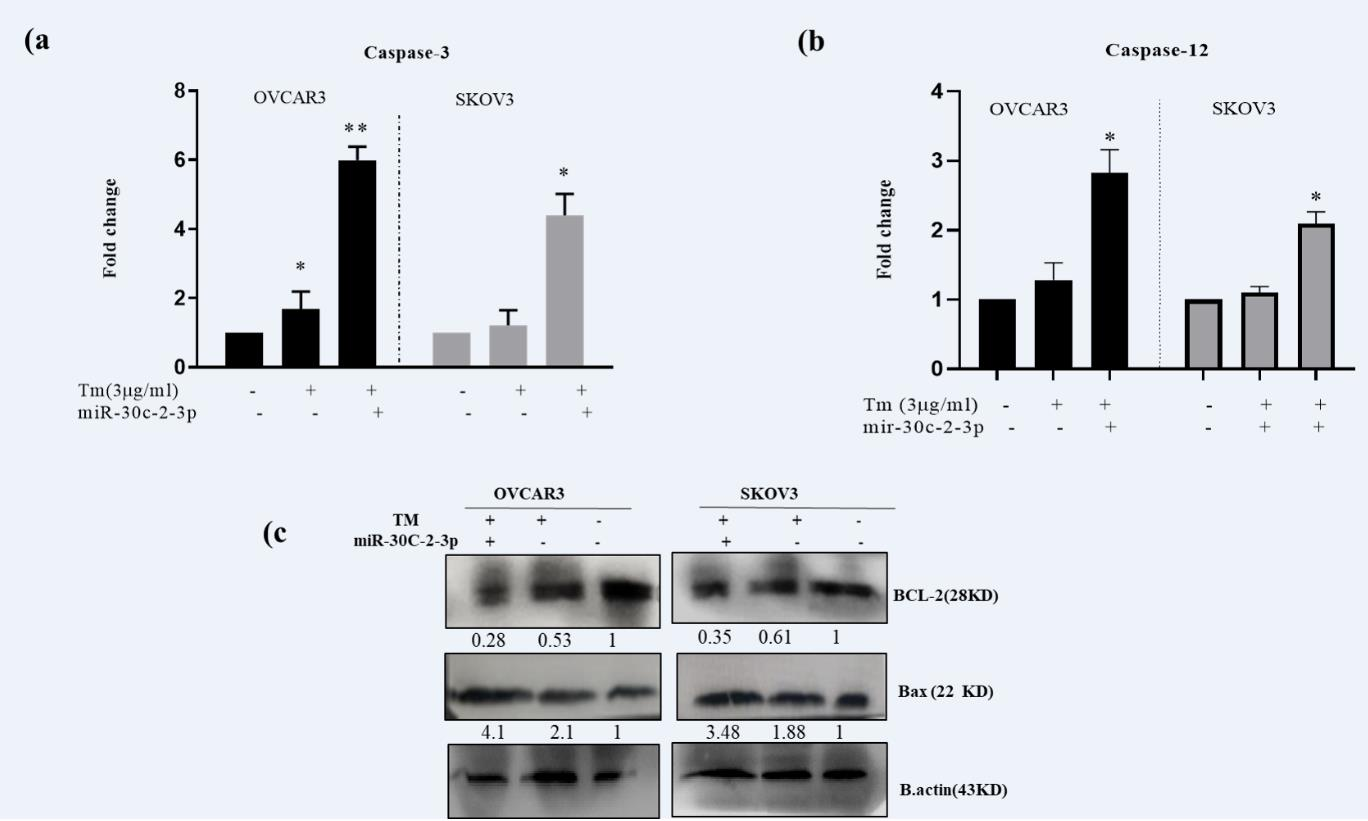
Figure 5: Transfection of miR-30c-2-3p enhanced caspase-3 and -12 activities and Bax/Bcl-2 protein levels during ER stress in ovarian cancer cells. OVCAR3 and SKOV3 were transfected by miR-30c-2-3p (treated with Tm for 18 h), then the apoptotic pathway was determined using (a, b) caspase-3 and -12 assay kits. Also, Bax/Bcl‐2 protein levels (c) were measured by Western blotting assay. Results were presented as mean ± SD and p<0.05 (*) and p<0.01 (**) considered as statistically significant.

To clarify the apoptotic mechanism of MicroRNA-30c-2-3p, we evaluated caspase-12 (a caspase related to ER) and -3 activities in both ovarian cancer cell lines during the ER stress. The cells were transfected with MicroRNA-30c-2-3p and treated with Tm for 18 hours and then separately compared to Tm-treated and scrambled control cells. Caspase-12 mediates the ER stress-apoptosis in rats (Bakhshi et al., 2008[4]) and human (Tungkum et al., 2017[38]). In our study, caspase‐12 and -3 activities were evaluated in transfected and non-transfected cells in response to the Tm treatment (Figure 5a(Fig. 5)). The results showed an increased level of caspase‐3 (5.99±0.37 fold in OVCAR3; P˂0.01 and 4.39±0.32 fold in SKOV3; P˂0.05) and caspase-12 (Figure 5b(Fig. 5), 2.82±0.34 fold for OVCAR3; P˂0.05 and 2.09 ±0.17 fold for SKOV3; P˂0.05), which were compared to Tm-treated cells. 

Additionally, we evaluated the expression of pro-apoptotic Bax and anti-apoptotic Bcl-2 proteins. As Figure 5c(Fig. 5) shows, the level of the Bax/Bcl-2 protein ratio was increased in transfected cells (p<0.01) than in Tm‐treated cells. These results indicated that degradation of XBP1 or damping of the sXBP1 translation by mimic MicroRNA-30c-2-3p increased apoptosis in SKOV3 and OVCAR3 cells, and restrained proliferation during the ER stress. MicroRNA-30c-2-3p enhanced apoptosis through high activation of caspases 12 and 3 as well as modulation of Bcl-2 and Bax protein expressions.

## Discussion

Ovarian cancer incorporates a mixed group of malignancies and differs from numerous aspects including molecular biology. Currently, different targeted treatment strategies and biological drugs have been developed to manage ovarian cancer. ER stress (ERS) and UPR signaling are a new target in controlling tumor growth. Solid tumor cells have hypoxia, nutrient deprivation and low pH microenvironment leading to ERS. These tumors are addicted to intact UPR signaling for survival, and XBP1 is a key adaptive protein in UPR (Giampietri et al., 2015[15]; Siwecka et al., 2019[32]; Wang and Kaufman, 2014[39]). Moreover, several studies have shown that XBP1 displays a role in the pathological aspect of ovary diseases. Importantly, the effect of XBP1 knockdown was the promotion of apoptosis and inhibition of cell cycle in the mouse granulosa ovary cells (Wang et al., 2017[41]). The XBP1 knockdown in the dendritic cell also caused dysfunction of anti-tumor immunity in human ovarian cancer cells (Song et al., 2018[34]). XBP1 is activated by the IRE1 RNase domain up-regulating the expression of various genes (Jin et al., 2016[17]). The blockage of IRE-XBP1 by chemical inhibitors is considered a therapeutic strategy. Since molecular therapy has lower toxicity than chemical drugs in the long term, and adaptable to each person, we used miR-30c-2-3p to target XBP1 in ovarian cancer cells. Additionally, microRNAs regulate various gene expressions, making them an ideal application for molecular therapy in cancer. In this study, we examined the effect of degradation of XBP1 by transfection of mimic miR-30c-2-3p on ovarian cancer cell lines. We transfected miR-30c-2-3p and then induced ERS with Tm to evaluate the apoptotic effect of miR-30c-2-3p on the cells. According to our results, the blockage of the XBP1 expression through miR-30c-2-3p promoted apoptosis in ovarian malignancy cells (Figure 3(Fig. 3)). Moreover, we evaluated the intensity of ERS in transfected cells undergoing the Tm treatment. The increased markers of ERS (CHOP and ATF4 mRNAs) and aggregated un/mis fold protein indicate that miR-30c-2-3p magnifies the severity of ERS, and promotes apoptosis (Figure 2(Fig. 2)). In our study, ERS apoptotic-associated CHOP and BIM proteins were modestly up-regulated, and the BIP protein was down-regulated (Figure 4(Fig. 4)). Furthermore, miR-30c-2-3p significantly increased the activity of caspases -12 and -3 (Figure 5a, b(Fig. 5)) and the Bax/BCL-2 ratio during the ER stress in transfected cells (Figure 5c(Fig. 5)). It is considerable that SKOV3 cells exhibited higher viability and resistance to the ER stress and the blockage of XBP1 compared to the OVCAR3 cell line. This difference may be due to genotypic and phenotypic differences in each cell line.

In this study, our results showed the potency of mimic miR-30c-2-3p in controlling the XBP1/s expression (Figure 1(Fig. 1)). Moreover, we revealed that transfection of mimic miR-30c-2-3p could affect the expression of CHOP, BIM and GRP78/BIP proteins. sXBP1 is a spliced form of XBP1 with transcription factor properties that promotes the expression of a variety of chaperons, especially BIP chaperon (Lee et al., 2003[20]). The level of BIP, as main regulator of the UPR, is crucial in the folding capacity of ER. GRP78/BIP is the most important chaperon in controlling the ER stress and is valuable in cancer therapeutics (Wang et al., 2009[40]). Over-expression of BIP is considered as a poor prognosis and involves in cisplatin resistance of ovarian cancer cells (Dong et al., 2008[13]; Li et al., 2014[22]). In the present study, the expression of the GRP78/BIP protein was mildly reduced by miR-30c-2-3p in transfected ovarian cancer cells, suggesting that the blockage of XBP1 through miR-30c-2-3p can enhance the accumulation of the mis/unfold protein and promote apoptosis. Therefore, we evaluated the protein aggregation by the ThT assay (fluorescence dye-binding mis/unfold proteins) and measured ER stress markers using RT-PCR in miR-30c-2-3p transfected cells. These results showed an accumulation of mis/unfold proteins and modest magnification of the ER stress by miR-30c-2-3p in both cell lines. Therefore, intense and uncontrolled ER stress appears to be responsible for triggering the programmed cell death. 

MTT and BrdU assays showed reduction of viability and inhibition of the cell growth in miR-30c-2-3p transfected cells. Moreover, the results of the flow cytometry test showed that transfection of mimic miR-30c-2-3p considerably induced apoptosis in ovarian cancer cells during the ER stress (Figure 3c(Fig. 3)). The data indicated the apoptotic and power anti-proliferative effects of miR-30c-2-3p by impeding the XBP1 expression. Similarly, reduction of XBP1 in pancreatic cancer and multiple myeloma cells by IRE1 inhibitors also exhibited toxicity (Chien et al., 2014[9]). Furthermore, Byrd et al. showed an apoptotic effect for miR-30c-2-3p in HeLa cells (Byrd et al., 2012[7]). Depletion of XBP1 induces apoptosis and inhibits tumor growth in other cell lines via various mechanisms. For example, XBP1 shRNA-treated MDA-MB-231 cells exhibit lower growth by controlling the HIF1α pathway (Chen et al., 2014[8]). Knockdown of XBP1 by RNAi in mouse granulosa cells induces apoptosis and inhibits the cell cycle through upregulation of CHOP, caspase-3 and Cyclin E (Wang et al., 2017[41]). In this regard*, *miR-30c-2-3p may influence various genes that can be involved in the apoptotic program; however, it needs more investigations into ovarian cancer cells. 

To further study the mechanism of apoptosis, we determined the levels of anti-apoptotic Bcl-2, pro-apoptotic Bax activities of caspases 3 and 12. miR-30c-2-3p significantly decreased Bcl‐2 and increased the Bax expression and caspase -12, -3 activities in both cell lines. Primarily, it is believed that activation of caspase-12 initiates the ERS-induced apoptosis (Szegezdi, 2003[35]). Some studies have concluded that human caspase-12 has only cytokine regulatory properties (Saleh et al., 2004[29]; Shiraishi et al., 2006[31]). However, several studies have shown the apoptotic function of caspase-12 in the cell lines affected by the drug-induced ERS (Bakhshi et al., 2008[4]; Gan et al., 2017[14]; Rao et al., 2001[28], 2002[27]). 

Moreover, we evaluated the BIM and CHOP pro-apoptotic proteins related to ERS. BIM is an important pro-apoptotic protein in UPR signaling induced through the CHOP-C/EBP-α protein (Puthalakath et al., 2007[26]; Tay et al., 2012[37]). The results showed the induction of CHOP and BIM in the presence of mimic miR-30c-2-3p in both cell lines during the ER stress. Similarly, transfection of mimic miR 200c-3p (Sohn, 2018[33]) can up-regulate ERS and be a potential target in the management of prostate cancer cells. Moreover, a decrease in XBP1s in pancreatic and multiple myeloma cancer cells showed an up-regulation of CHOP in response to IRE1α-RNase inhibitors (Chien et al., 2014[9]; Mimura et al., 2012[24]). However, Byrd et al. have reported that miR-30c-2-3p exhibits regulatory effects on CHOP in overexpressed miR-30c-2-3p HeLa cells ( Byrd et al., 2012[7]). These diversities may be due to differences in the model study, or in molecular characteristics and complexities among cell lines. Overall, our results showed that mimic miR-30c-2-3p had mild regulatory effects on the UPR signaling, and considerably reduced the XBP1/s expression in ovarian malignancy cells.

## Conclusion and Future Prospects

The current study demonstrates that XBP1 plays a crucial role in the ERS-induced ovarian cancer apoptosis. Our data indicate that miR-30c-2-3p impedes the expression of XBP1/s, causing reduced viability of ovarian malignancy cells. The ability of micro RNA-30c-2-3p to reduce XBP1/S and BIP/ GRP78 augments the intensity of ERS. This magnification of ERS increases ER-specific caspase-12 and -3 activities and pro-apoptotic CHOP and BIM protein expressions. Many studies have revealed that BIP overexpression is a poor prognosis in several malignancies, particularly ovarian cancer. Moreover, these findings suggest that mimic miR-30c-2-3p can be considered a strategy to reduce the growth of ovarian malignancy cells. However, it is necessary to conduct an *in vivo* study to evaluate the long-term effects of miR-30c-2-3p.

## Acknowledgement

This project was in part financially supported by Isfahan University of Medical Sciences (Grant Number: 396511). Also we used Doc print CX5 Gel Documentation System (Vilber Lourmat) to produce digital images of Western blotting in Iran University of Medical Sciences, Tehran.

## Authors’ contribution

MA, SRB and AM designed the experiments. SRB performed the experiments. SRB, MA analyzed the results. SRB performed MTT, BrdU, Flow-Cytometry, Western blotting, staining and imaging. MA, SRB wrote the manuscript. All authors have read and approved the final version of the manuscript.

## Conflict of interest

The authors declare that they have no known competing financial interests or personal relationships that could have appeared to influence the work reported in this paper.

## Informed consent

The authors declare that this article does not contain any studies with human participants and patients.

## Figures and Tables

**Table 1:**
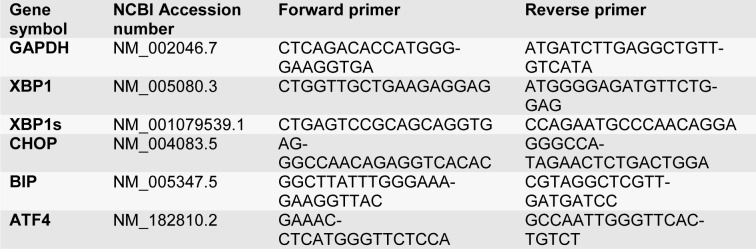
Primer sequences of ER stress gene were used for real-time RT‐PCR
